# Assessing the anticipated growth response of northern conifer populations to a warming climate

**DOI:** 10.1038/srep43881

**Published:** 2017-03-07

**Authors:** John H. Pedlar, Daniel W. McKenney

**Affiliations:** 1 Natural Resources Canada, Canadian Forest Service, Great Lakes Forestry Centre, 1219 Queen Street East, Sault Ste. Marie, Ontario, P6A 2E5, Canada

## Abstract

The growth response of trees to ongoing climate change has important implications for future forest dynamics, accurate carbon accounting, and sustainable forest management. We used data from black spruce (*Picea mariana*) and jack pine (*Pinus banksiana*) provenance trials, along with published data for three other northern conifers, to identify a consistent growth response to climate warming in which cold-origin populations are expected to benefit and warm-origin populations are expected to decline. Specifically, populations from across the geographic range of a species appear to grow well at temperatures characteristic of the southern portion of the range, indicating significant potential for a positive growth response to climate warming in cold-origin populations. Few studies have quantified and compared this pattern across multiple species using provenance data. We present a forest regeneration strategy that incorporates these anticipated growth responses to promote populations that are both local to the planting site and expected to grow well under climate change.

The response of forests to climate change has far-reaching ecological and economic implications. Besides conventional forestry operations, which contribute approximately US$100 billion annually to the global economy^1^, forests have been estimated to provide trillions of dollars in ecosystem services^2^. For instance, global forests sequester approximately 3 billion tons of carbon each year^3^ and provide habitat for roughly 65 percent of all terrestrial species^4^. The boreal forest, which is distributed extensively across high latitudes in the northern hemisphere, contributes significantly to these global numbers – accounting for approximately 32% of global terrestrial carbon stocks and about 30% of global lumber and paper markets^5^, and providing essential habitat for a diverse range of plants and animals^6^. Understanding the response of these northern forests to climate change is important for projecting future forest dynamics, determining ongoing sustainable harvest levels, and making reasonable projections of climate change impacts on carbon cycling and related ecosystem services.

Many approaches have been employed to investigate tree growth responses to climate change, including tree ring analyses^7^,^8^, forest regeneration studies^9^, and open-air CO2 experiments^10^. Provenance trials represent another useful data source for addressing this topic. These studies, which have been carried out for centuries^11^, involve planting seeds gathered from a variety of populations (also referred to as provenances or seed sources) at a series of test sites (or common gardens). Large-scale provenance studies – which may involve hundreds of seed sources planted in various combinations across tens of test sites – are relatively rare, as they require significant amounts of planning, cooperation, and resources in order to establish, maintain, and measure them through time. Though originally designed with tree breeding and production forestry in mind, these experiments have been used to explore tree growth and seed movements under climate change^12^,^13^,^14^,^15^. However, studies to date have tended to focus on single species relationships, thus insights into broader patterns that could apply across taxonomic groups have generally been lacking.

Historically, provenance trial data have been analyzed using either transfer or response functions^16^. In the case of transfer functions, a response variable (e.g., height of each seed source at a certain age) is modelled as a function of the distance of each seed source (often measured in climatic units) from the test site. In contrast, response functions portray how a single seed source trait (e.g., height) varies across test sites with respect to a climate variable of interest. Recent efforts have extended the basic transfer and response function approaches. O’Neill *et al*.^16^ presented a universal transfer function that fits a separate transfer function for each test site and then fits a relationship between these site-level parameters (i.e., slopes and intercepts) and site climate. Wang *et al*.^17^ presented a method that essentially combines the response and transfer function approaches described above into a universal response function that can predict the height growth of any seed source at any planting site within the range of the data used to develop the relationship. Finally, Leites *et al*.^18^ proposed merging species- and population-level responses into one model using mixed-effects modeling techniques. Here we make use of traditional response functions as they allow our findings to be conveyed in a relatively simple and transparent manner.

We employ a combination of provenance trial data and published data/findings to examine the anticipated growth response of five northern conifers to climate change. Our key finding is remarkably consistent across these species, with cold-origin (northern) populations expected to exhibit a positive growth response to a significant amount of climate warming, while warm-origin (southern) populations are expected to exhibit near-immediate declines. We conclude by exploring the implications of this finding for tree growth under climate change and identify an approach that explicitly incorporates these considerations into forest regeneration and restoration efforts. This work provides several insights that have been largely overlooked, despite their significant implications for forest dynamics under rapid climate change.

## Results

**Figure 1:**
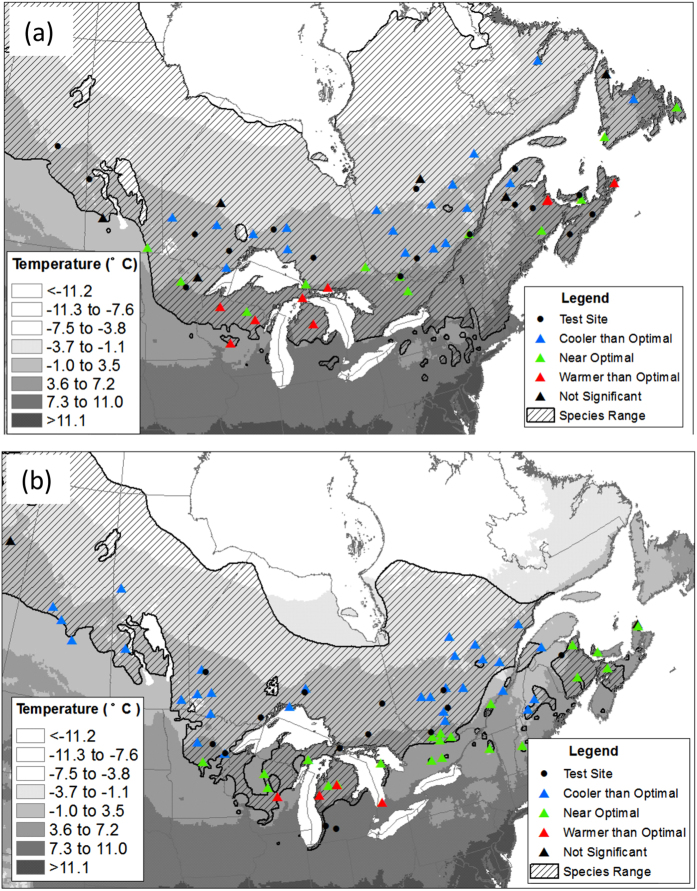
Test sites (circles) and seed sources (triangles) used in (**a**) black spruce and (**b**) jack pine provenance trials. Differentiated on the map are populations growing at temperatures >1 °C cooler than optimal (blue triangles), within ± 1 °C of optimal (green triangles), and >1 °C warmer than optimal (red triangles). Also shown is the spatial distribution of mean annual temperature values and the geographic range of each species (hatching). Maps were drawn using ARCGIS v.9.3 (ESRI, Redlands, CA, USA; http://www.esri.com/arcgis/about-arcgis).

**Figure 2:**
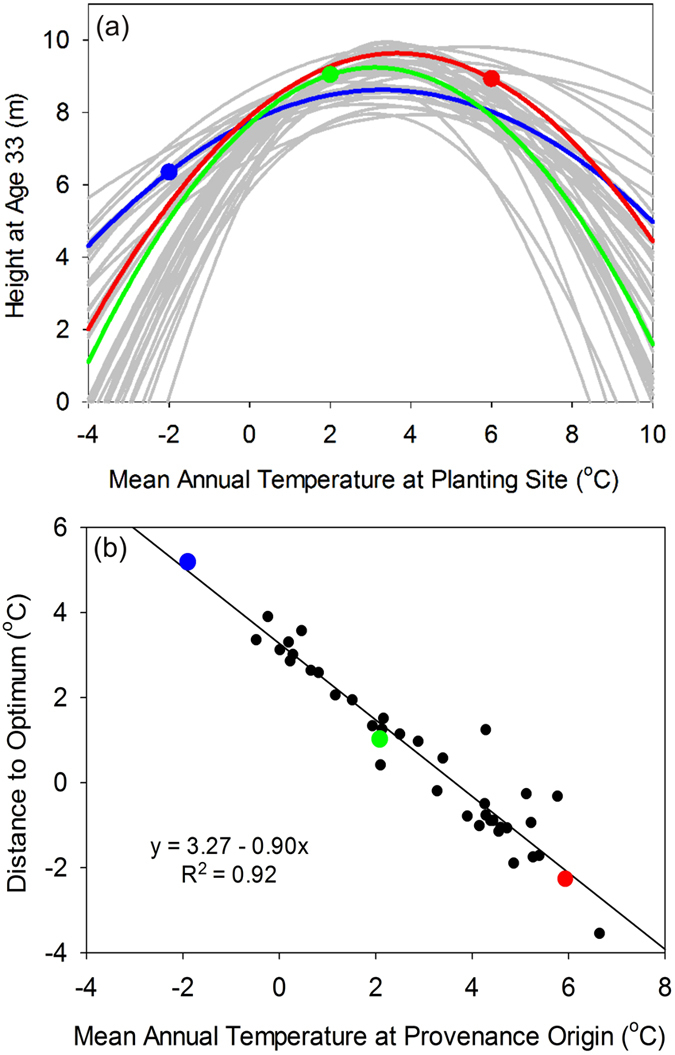
Provenance data summaries for black spruce showing: (**a**) response curves for 39 seed sources (gray), including representative seed sources originating from warm (red), cold (blue), and moderate (green) locations; and (**b**) distance to optimal MAT for 39 seed sources (black dots), highlighting the same representative seed sources as those shown in Fig. 2a.

Forty-five black spruce seed sources were included in the analysis (Fig. 1a; see Methods for details). Thirty-nine (87 percent) of these seed sources exhibited significant bell-shaped height growth responses to mean annual temperature (MAT) at the planting site (Fig. 2a; see Fig. S1 for individual population plots). For these populations, MAT explained a significant proportion of the variation in height growth, with an average coefficient of determination (R^2^) of 48.2%. The average of the population-specific optimal MAT (MAT_opt_ ± S.E.) values was 3.56 ± 0.10 °C (Table 1). The remaining six populations also exhibited bell-shaped curves, but were not statistically significant (i.e., p-values were greater than 0.2). There are many factors that may confound response function relationships, including both within- and between-test site variation in: edaphic conditions, herbivory and disease impacts, management practices, extreme climate events, and tree-level genetic composition. Given these many extraneous influences, we contend that these results provide strong evidence for a temperature-growth relationship that is consistent across most black spruce populations.

Highlighted in Fig. 2a are representative populations originating from warm (red), moderate (green), and cool (blue) environments. Note that the peak of the respective growth curves is in: cooler environments for the warm-origin population, warmer environments for the cool-origin population, and local environments for the population of moderate temperature origin. Note also that the warm-origin seed source has the highest growth potential (i.e., reaches maximum height), while the cool-origin seed source has the lowest. The spatial distribution of cool-origin, warm-origin and near-optimal seed sources is shown in Fig. 1a. Climatic distance to MAT_opt_ (CD_opt_) was calculated for all populations and plotted against MAT at population origin (Fig. 2b). This produced a declining linear relationship that was highly significant, had low parameter error estimates, and explained the majority of variation in CD_opt_ (Table 1). This finding provides strong evidence that cold-origin populations are growing under cooler than optimal conditions, while warm-origin populations are growing under warmer than optimal conditions.

**Figure 3:**
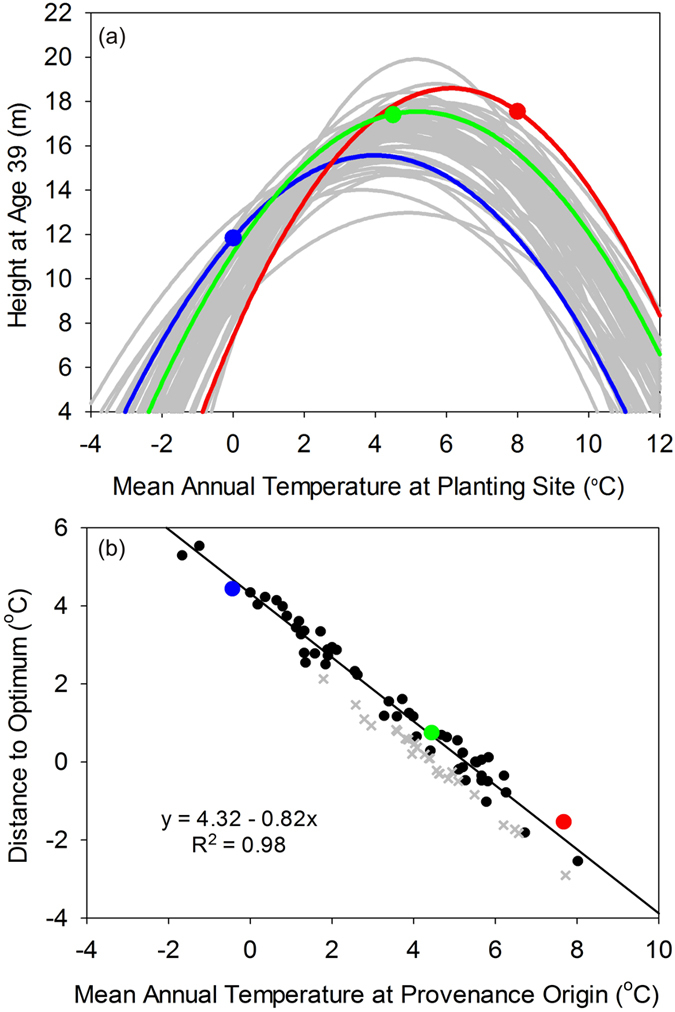
Provenance data summaries for jack pine showing: (**a**) response curves for 44 seed sources (gray lines), including representative seed sources originating from warm (red), cold (blue), and moderate (green) locations; and (**b**) distance to optimal MAT for the same 44 seed sources (black dots) and representative seed sources (colored dots), as well as for 26 seed sources from an independent jack pine provenance dataset (gray x’s).

Fifty-seven jack pine seed sources were included in the analysis (Fig. 1b; see Methods for details). Fifty-five (96 percent) of these seed sources exhibited a significant bell-shaped height growth response to MAT at the planting site (Fig. 3a; see Fig. S2 for individual seed source plots), with an average MAT_opt_ ( ± S.E.) value of 4.93 ± 0.07 °C (Table 1). MAT explained a significant proportion of the variation in these relationships (average R^2^ = 50.0%). Similar to the black spruce findings described above, warm-origin jack pine populations tended to grow best under cooler environments (see red features in Figs 1b and 3a), cool-origin populations tended to grow best under warmer environments (see blue features in Figs 1b and 3a), and moderate-origin populations grew best locally (see green features in Figs 1b and 3a). Warm-origin seed sources again exhibited the highest growth potential, while the cool-origin seed source had the lowest. When CD_opt_ was calculated for all populations and plotted against MAT at population origin (Fig. 3b), a highly significant negative linear relationship was observed (Table 1) – again providing compelling evidence that cool-origin populations are growing under cooler than optimal conditions, while warm-origin populations are growing under warmer than optimal conditions.

MAT_opt_ and CD_opt_ were also calculated for each seed source in an independent jack pine dataset. Despite covering a narrower range of environmental conditions than the rangewide trial, all 26 seed sources exhibited bell-shaped growth response curves in relation to planting site MAT (average R^2^ = 49.4%), with an average MAT_opt_ ( ± S.E.) value of 4.41 ± 0.05 °C. CD_opt_ values, calculated for each seed source in the independent dataset, were well predicted by the linear regression from the rangewide trial that related CD_opt_ and MAT_i_ (Fig. 3b). The mean absolute prediction error for CD_opt_ was 0.71 °C, with a bias of −0.71 °C; indicating that the predictions were similar to, but consistently larger than, the calculated values (see location of trend line relative to independent data points in Fig. 3b).

**Figure 4:**
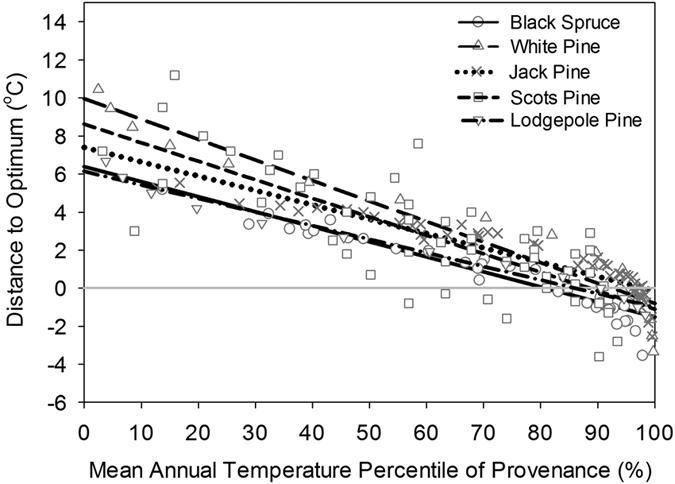
Relationship between distance to optimal MAT and MAT at provenance origin (expressed as a percentile relative to the range of MAT values occupied) for five northern conifer species.

By converting MAT at seed source origin to a percentile value expressed relative to the range of MAT values occupied by a species (i.e., MAT percentile), we were able to display the distance to MAT_opt_ relationship on the same graph for the two species described above and for three additional tree species – white pine (*Pinus glauca*), lodgepole pine (*Pinus contorta*), and Noway spruce (*Pinus sylvestris*) – for which population-specific MAT_opt_ information was obtained from the scientific literature (Fig. 4). All five species exhibited strikingly similar growth preferences, with local temperatures becoming increasingly cooler than optimum towards the cool end of the temperature range occupied by each species and increasingly warmer than optimum towards the warm end of the occupied temperature range. Local MAT conditions appeared ideal for growth at MAT percentiles between approximately 85–95% for all species (Fig. 4); in fact, temperatures in this range supported relatively high growth rates for nearly all seed sources, regardless of MAT at provenance origin.

## Discussion

Our findings indicate that the growth response of northern conifers to climate warming will be strongly influenced by the position of a population within the temperature profile occupied by a species. Specifically, populations at the warm end of the profile (southern edge of the range) are expected to decline, while those at the cool end of the profile (northern portion of the range) are expected to benefit from at least a certain amount of climate warming. Clearly there are many climate change-related factors beyond average temperature that may influence future tree growth. For example, changes in climate extremes (e.g., drought), disturbance regimes (e.g., insects and fire), and water-use efficiency due to CO_2_ fertilization have been identified as factors shaping the structure, composition, and productivity of future forests^7^,^8^,^19^,^20^,^21^. While these factors add considerable complexity to growth projections, we feel there is significant value in establishing a baseline expectation regarding tree response to warming temperature – a key driver of growth in northern forests^22^.

Our findings appear to be relatively robust both within and between species. The MAT-based quadratic regressions produced bell-shaped response functions that explained a significant amount of variation in height growth for the majority of seed sources. Furthermore, all species exhibited strong relationships between CD_opt,_ and MAT, with low parameter error estimates, and a consistent preference for temperatures characteristic of the southern portion of the range (i.e., between the 85^th^ and 95^th^ MAT percentile). Finally, the relatively small prediction errors – estimated using the regional jack pine provenance dataset – provide confidence that the relationships developed here are applicable to a variety of regions and forest ages. The reason for the modest bias in the prediction errors is not clear, but may be associated with subtle regional differences in MAT preferences.

Certain aspects of this work have been reported previously. For instance, several studies have recognized that southern edge populations are at heightened risk of suffering climate change-related declines^23^,^24^,^25^,^26^, while other studies have projected a positive growth response by northern populations^27^. The superior growth potential of southern and central seed sources has also been reported previously^12^,^26^,^28^,^29^. Namkoong^30^ reported that seed sources are not necessarily optimally adapted to their local environments – citing numerous instances where seeds performed better under warmer growing conditions. A number of provenance studies have reported similar response function patterns as those presented here – but for single species and often without explicitly recognizing the broader implications for growth under climate change^12^,^13^,^31^,^32^. To our knowledge, the current study is the first to employ provenance data to elucidate a consistent growth response to climate warming for a variety of northern conifers.

Reich *et al*.^33^ examined the photosynthetic and growth responses of 11 species of juvenile trees to experimental above- and belowground warming at a study site in northern Minnesota. They reported that boreal species (for which the study site fell at the southern edge of their geographic ranges) responded negatively to the experimental warming, while temperate species (for which the study site fell at the northern edge of their geographic ranges) responded positively. Thus, our findings are consistent with those of Reich *et al*.^33^ despite widely differing experimental approaches. In fact, the two studies provide a number of complementary findings that collectively strengthen the argument that a population’s position within the overall temperature profile of a species plays an important role in tree response to climate change. For instance, Reich *et al*.^33^ employed ecologically realistic densities and species mixtures at their study plots, which led them to conclude that these temperature position effects operate in the face of interspecific competition. Conversely, the provenance trial data employed here are from single-species plantations; however, the long-term nature of the provenance studies (up to 39 years compared to 3 years in Reich *et al*.^33^) suggests that these effects may endure for many decades. Furthermore, the range-wide nature of the provenance data allowed us to elucidate the response of populations across a full gradient of temperatures, which was not possible based on the single study site employed in Reich *et al*.^33^. Our findings are further supported by studies that have demonstrated temperature-driven growth increases in northern conifer populations using both dendrochronological^22^,^34^,^35^ and satellite-based^36^,^37^ lines of evidence; though these temperature-driven responses may be overshadowed by water limitations in some boreal regions^7^,^38^,^39^.

Why does this consistent pattern exist across these species? The phenotype (i.e., height growth in this case) expressed at a given location is strongly influenced by local conditions and gene flow. Trees are known to have high levels of gene flow, which is thought to limit the extent to which populations can become highly adapted to local conditions^25^. Garcia-Ramos and Kirkpatrick^40^ hypothesized that genes flow outward from highly abundant and fecund populations at the range center to peripheral populations. As a result, peripheral populations exhibit a migration load, wherein average fitness is reduced due to the presence of maladaptive immigrant alleles. Importantly for growth response under climate change, populations south of range center would be expected to contain genotypes that are better suited to colder conditions, while northern populations would be expected to respond positively to warmer conditions^25^. This hypothesis largely explains the patterns observed here; however, our analysis indicated that the temperature preferences of these species lie significantly south of their current climatic range centers (i.e., between the 85^th^ and 95^th^ MAT percentile). We propose that this pattern may be related to the glacial history of these species – wherein northward expansions have proceeded from southern glacial refugia^41^,^42^. Despite millennia of migration and local adaptation since the last glacial retreat in North America, it may be that these southern genetic origins continue to exert a strong influence over the climate preferences of contemporary populations.

Response functions, such as those presented here, illustrate the phenotypic plasticity – i.e., altered characteristics in response to environmental change – of the populations under study. This type of response is particularly important in the context of climate change because it represents a nearly immediate adjustment that populations could make to rapidly changing environmental conditions. In contrast, due to long generation times, a genetic response to climate change by trees is expected to lag significantly behind the pace of climate change^27^. Furthermore, given natural migration rates of 10–50 km per century^43^, northern hemisphere tree species would require many centuries to track projected climate envelope shifts – which may be up to 700 km during this century^44^. Thus, the response of tree species *in situ* over the next 100 years is likely to be largely dependent on plastic responses such as those presented here.

Our findings may contribute to efforts that require accurate projections of forest growth and dynamics under climate change. For example, timber supply analyses require projections of future forest growth in order to set sustainable harvest levels and identify forest stands for both short- and long-term harvest activities^45^. The growth patterns described here could contribute to modifying regional growth and yield projections, which would in turn drive changes in timber supply model outputs. Furthermore, forest vegetation models employ information on species’ traits and relative growth rates to predict changes in species composition over time^46^. Again, the information provided here may help investigators to better understand species dynamics under climate change – particularly at range boundaries where transitions are likely to occur. Note that Reich *et al*.^33^ employed similar findings to predict the expansion of three temperate species and decline of two boreal species at a test site in northern Minnesota.

These findings also have implications for climate change adaptation efforts in the context of forest regeneration and restoration. Assisted migration of tree populations and/or species has been proposed as an approach for adapting future forests to climate change^47^,^48^,^49^. This approach has generated considerable debate within the conservation community as it challenges the longstanding paradigm of managing species and populations within traditional range limits. Based on the concepts advanced here, a low risk alternative could be achieved by incorporating anticipated growth responses into forest regeneration and restoration efforts. Specifically, at a given planting site, the pool of local species (i.e., those species with ranges that overlap the planting site) could be ranked according to the climatic distance between the planting site and each species’ 90^th^ MAT percentile. In this way, species for which the planting site falls in the northern portion of the range could be identified as candidates for reforestation at that location. The climate profile information required to carry out such an analysis is available for thousands of North American plant species at http://planthardiness.gc.ca/. Unlike assisted migration, this approach can be carried out without moving species – or even populations – outside natural limits. Consequently, it has the potential to address several management objectives at the same time – namely it would establish species and/or populations that are both native to the planting area and expected to exhibit a positive growth response as climate warms. The potential impact of such an approach would be greatest in temperate regions and along the boreal-temperate forest ecotone, where relatively high levels of tree species richness would allow for a variety of species selection options.

There are a number of caveats to employing our findings in this manner. Our current work is focused on northern conifer species, which clearly limits the scope of the concepts advanced here. While there is some evidence that populations of temperate, broad-leaved tree species exhibit similar responses to climate warming^31^,^33^, understanding how anticipated growth responses vary across geographic regions and taxonomic groups will be the focus of ongoing research. In this regard, we note that white pine – though having a geographic distribution that extends well into the southeast region of the United States – exhibited a response that was entirely consistent with the four strictly boreal species in this study. Furthermore, a distinction should be drawn between the anticipated positive growth response of northern populations to climate warming and the optimal growth response at a given location. As noted above, southern populations have been recognized as having superior growth attributes relative to northern populations; thus, despite improved growth by northern populations under climate change, the assisted migration of southerly seed sources may be required if maximum productivity is desired^27^. In fact, given the wide range of management objectives and the significant uncertainty surrounding climate change projections, the approach advanced here is best viewed as one of several complementary tools – including assisted migration – that could be used to identify climatically appropriate planting stock for forest regeneration and restoration efforts.

## Materials and Methods

### Overview

We employed a number of datasets, described in detail below, to elucidate the anticipated growth response of five northern coniferous tree species to climate change. For black spruce and jack pine, we used provenance trial data to generate a climatic response function for each seed source. In the case of jack pine, we also obtained a second source of provenance data, allowing us to test the predictive accuracy of the original regression models on an independent dataset. For the remaining species, information on climatic preferences was obtained from published equations and figures. Finally, we used information from an online repository of information on plant distributions and related climatic associations to express temperature at each seed source origin as a percentile value; this allowed us to directly compare the temperature preferences of the five tree species under study.

### Provenance and Climate Data

Black spruce provenance data was obtained from remeasurements on a portion of the Canadian Forest Service’s (CFS) long-term black spruce provenance trial, which originally incorporated 202 seed sources across 34 test sites in Canada and the United States (see Selkirk^50^ for details). The remeasurements were carried out in 2003 (33 years of age from seed) and involved measuring height and DBH of all surviving trees at each test site (see Thomson *et al*.^26^ for details). In total, 192 provenances at 18 test sites in Canada and one test site in Minnesota were measured.

Jack pine provenance data was obtained from remeasurements on a portion of the CFS 255 Series rangewide jack pine provenance trial, which consisted of 99 seed sources planted in various combinations at test sites across eastern Canada, the United States, and Europe (see Rudolph and Yeatman^51^ for details). During the summer of 2005, at 39 years of age from seed, all 16 remaining viable test sites in Canada and the United States were remeasured (see Thomson and Parker^28^ for details).

A second jack pine provenance dataset was obtained from a trial that covered the Great Lakes region of the United States and included 26 seed sources and seventeen test sites (see Jeffers and Jensen^52^ for a map of the study area and further details). This trial was remeasured in 1973 (at 20 years of age from seed) and average seed source height values at each test site were made available (see Table 4 in Jeffers and Jensen^52^). Given the relative scarcity of provenance data, this second source provided a rare opportunity to test the predictive accuracy of the relationships developed using the rangewide jack pine provenance data described above.

We obtained information regarding seed source-specific growth optima in relation to climate for three other tree species from the scientific literature. For white pine (*Pinus glauca*), we employed the universal response function (URF) provided in Table 1 of Yang *et al*.^53^ to estimate the optimal climate for a number of hypothetical seed sources (see Table S2 for worked example). Note that URFs, originally developed by Wang *et al*.^17^, model tree growth as a function of both the planting site climate and seed source climate and thus, in principle, allow the growth of any seed source to be estimated at any location. Similarly, we employed a published URF (see Table 2 in Wang *et al*.^17^) to estimate optimal climate values for a range of hypothetical lodgepole pine (*Pinus contorta*) seed sources (see Table S3 for worked example). Finally, optimal climate information for 54 Scots pine (*Pinus sylvestris*) populations was obtained directly from Fig. 6a in Rehfeldt *et al*.^32^.

Estimates of mean climate were obtained by interrogating spatial models of 1961–1990 normals at the location of each provenance and planting site. These models were developed by interpolating temperature and precipitation data from over 12,000 stations from across Canada, Alaska and the contiguous United States (see McKenney *et al*.^54^ for details). The interpolations were carried out using ANUSPLIN^55^, which makes use of thin plate smoothing splines to develop spatially continuous climate models that allow for robust determinations of historical climate estimates at any location where latitude, longitude and elevation are known. Eight climate variables were explored as potential drivers of tree height growth, including: mean annual temperature (MAT), average daily maximum temperature of the hottest month (MAXT), average daily minimum temperature of the coldest month (MINT), annual extreme minimum temperature (XMINT), annual precipitation (PREC), precipitation of the three coldest months (PRECCQ), precipitation of the hottest three months (PRECHQ), and an annual climate-moisture index (CMI) – which provides an estimate of water balance in the absence of soil considerations (sensu Hogg *et al*.^56^).

### Statistical Analysis

For black spruce and jack pine, we modelled tree height as a function of climate at the planting site using a separate quadratic function for each seed source:


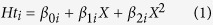


where Ht_i_ is the height of seed source *i*, X is the value of the climate variable at a planting site, and the β’s are the fitted parameters. Preliminary analyses indicated that, of the eight climate variables examined, MAT was the strongest predictor of height growth for black spruce and the second strongest predictor (narrowly behind MAXT) for jack pine (Table S1). Given that MAT was a strong predictor for both species and has been identified as an important driver of forest genetic variation in previous studies^12^,^17^,^18^,^53^, we focus on MAT for the remainder of this study.

We employed quadratic functions in order to capture the anticipated Gaussian (i.e., bell-shaped) relationship between tree growth and climate – a pattern that has been demonstrated in previous provenance studies^13^,^32^. While other bell-shaped functions have been employed (e.g., the Cauchy function^26^), we opted for the quadratic function as it allowed significant flexibility in the shape of the fitted curves (ranging from parabolic to flat) and, on visual inspection, fit the data reasonably well (Figs S1 and S2). For both species, we limited the seed sources included in the analysis to those that had been planted at 10 or more test sites, spanning a significant gradient of MAT (>6 °C) and PREC (>500 mm) conditions. These filters, which helped to ensure the quality and reliability of the regression results, reduced the number of seed sources in the analysis to 45 (from 192) for black spruce and 57 (from 99) for jack pine. Through visual inspection of residuals and assessment of Studentized residual scores, we identified several test sites that consistently behaved as outliers in preliminary analyses. As a result, the following three test sites were removed from further analyses: Roddickton, Newfoundland; Bald Mountain, New Brunswick; and Fraserdale, Ontario. Growth at these sites may have been impacted by soil or other site-level factors that were outside the broad climatic focus of the current study. The removal of these sites did not change the qualitative findings of the study, though it did improve the statistical fit of the data for certain seed sources.

The optimal MAT (MAT_opt_) for growth of each seed source (*i*) was calculated by taking the first derivative of each seed source’s fitted quadratic equation, setting it equal to zero and solving:


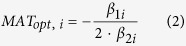


The difference between MAT_opt_ and MAT at seed source origin (MAT_i_) was calculated to quantify the climatic distance of each seed source (CD_opt_) to its optimal growing temperature:


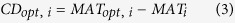


For each species (black spruce and jack pine), we then generated a graph and linear regression model describing the relationship between CD_opt_ and MAT_i_. These same steps (i.e., fitting seed source-specific response curves, and calculating MAT_opt,_ and CD_opt_) were repeated using the independent jack pine provenance data, allowing us to test the predictive accuracy of the linear regression model relating CD_opt_ and MAT_i_ for this species.

**Figure 5:**
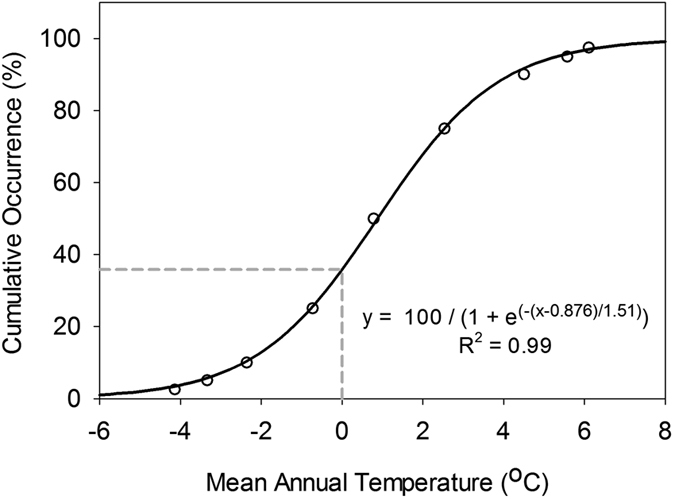
A cumulative occurrence curve for black spruce in relation to mean annual temperature. The data points were obtained from a North American plant occurrence database (http://planthardiness.gc.ca/) and were fit using a sigmoidal curve. As an example, the gray dotted line indicates the MAT percentile associated with a MAT value of 0 °C.

For the remaining species (white pine, lodgepole pine, and Scot’s pine), the data collected from the literature would have allowed us to create separate plots/models relating CD_opt_ and MAT_i_. However, in order to highlight cross-species similarities in this relationship, we elected to display all species on the same graph. In order to do this, MAT_i_ values were converted to percentiles, which indicated where a seed source was located relative to the range of MAT values occupied by a species. The data required to make this conversion were obtained from an extensive database of North American plant occurrence observations (http://planthardiness.gc.ca/; see McKenney *et al*.^57^ for details). These data have been used to generate climatic summaries (i.e., min, max, mean and percentiles) for more than 3000 North American plant species in relation to a wide variety of climate variables (including MAT). From these climate summaries, we generated a cumulative occurrence curve for each species in relation to MAT and then fit a sigmoidal equation to the relationship. Seed source MAT values were substituted into the species-specific sigmoidal equation to generate the associated percentiles (Fig. 5). Scots pine (native to Scandinavia) was not well-represented in the North American plant occurrence database described above; therefore, a cumulative occurrence curve was generated by sampling MAT at a 10-km resolution^58^ across the native Eurasian range of Scots pine (obtained from http://www.euforgen.org/distribution-maps/).

### Data Availability

All data are available at the Open Science Framework (https://osf.io/) with accession number: 10.17605/OSF.IO/72UZF.

## Additional Information

**How to cite this article**: Pedlar, J. H. and McKenney, D. W. Assessing the anticipated growth response of northern conifer populations to a warming climate. *Sci. Rep.* **7**, 43881; doi: 10.1038/srep43881 (2017).

**Publisher's note:** Springer Nature remains neutral with regard to jurisdictional claims in published maps and institutional affiliations.

## Supplementary Material

Supplementary Material

## Figures and Tables

**Table 1:** Parameters from linear regression analyses relating climatic distance from optimal growing temperature to mean annual temperature (MAT) at seed source origin for black spruce and jack pine.

Species	Intercept		Slope		P	R^2^	N	Optimum MAT^a^	
	Value	S.E.	Value	S.E.				Mean	S.E.
Black spruce	3.27	0.17	−0.90	0.05	<0.0001	0.92	39	3.56	0.10
Jack pine	4.32	0.15	−0.82	0.04	<0.0001	0.98	55	4.93	0.07

^a^Mean and standard error of the population-specific optimum MAT values for each species.
