# Supplementary material for: Assessing the anticipated growth response of northern conifer populations to a warming climate
Source: Sci Rep. 2017 Mar 7;7:43881. doi: 10.1038/srep43881 (PMC5339688; doi:10.1038/srep43881)

Supplementary Material

Assessing The Anticipated Growth Response Of Northern Conifer Populations To A Warming Climate

John H. Pedlar and Daniel W. McKenney

Table S1. Summary of quadratic regression results for various climate variables in explaining height growth of black spruce and jack pine seed sources.

Climate Variable[a]	Black Spruce		Jack Pine	
	Number of Populations [b] (%, in brackets)	Average Coefficient of Determination (R^2)	Number of Populations [b] (%, in brackets)	Average Coefficient of Determination (R^2)
MAT	39 (87)	0.48	55 (96)	0.50
MAXTHM	36 (80)	0.35	51 (88)	0.37
MINTCM	1 (2)	0.40	0 (0)	-
XMINT	0 (0)	-	0 (0)	-
PREC	27 (60)	0.09	19 (33)	0.31
PRECCQ	34 (76)	0.07	3 (5)	0.31
PRECHQ	12 (27)	0.11	10 (17)	0.38
CMI	16 (36)	0.07	1 (2)	0.35

[a] MAT = mean annual temperature; MAXTHM = average daily maximum temperature of the hottest month; MINTCM = average daily minimum temperature of the coldest month; XMINT = annual extreme minimum temperature; PREC = annual precipitation; PRECCQ = precipitation of the three coldest months; PRECHQ = precipitation of the three hottest months; and CMI = climate-moisture index.

[b] Number of populations showing a bell-shaped response curve when planted at 10 or more test sites that spanned a gradient of MAT (>6˚C) and PREC (>500 mm) conditions.

Table S2. White Pine height values (at age 16) for a range of mean annual temperatures (˚C) at seed source origin (MAT_{ss}) and planting site (MAT_{ps}), calculated from a published universal response function (Table 1, Yang et al. 2015): $Ht_{16} = -4.468 + 1.942 \cdot MAT_{ps} + 0.270 \cdot MAT_{ss} - 0.093 \cdot MAT_{ps}^2 - 0.022 \cdot MAT_{ss}^2 + 0.001 \cdot MAT_{ps} \cdot MAT_{ss}^2$.

MAT_{ss} → / ↓ MAT_{ps}	2	4	6	8	10	12	14
4	2.3	2.6	2.8	2.8	2.7	2.5	2.1
6	4.3	4.7	4.9	5.0	4.9	4.8	4.5
8	5.6	6.0	6.2	6.4	6.4	6.3	6.2
10	6.1	6.5	6.8	7.0	7.2	7.2	7.1
12	5.9	6.4	6.7	7.0	7.1	7.2	7.3
14	5.0	5.4	5.8	6.1	6.4	6.6	6.7

Table S3. Lodgepole Pine height values (at age 20) for a range of mean annual temperatures (˚C) at seed source origin (MAT_{ss}) and planting site (MAT_{ps}), calculated from a published universal response function (Table 2, Wang et al. 2010):

$$Ht_{20} = -20.07 + 2.006 \cdot MAT_{ps} + 0.223 \cdot MAT_{ss} - 0.294 \cdot MAT_{ps}^{2} - 0.039 \cdot MAT_{ss}^{2} - 0.077 \cdot MAT_{ps} \cdot MAT_{ss} + 0.031 \cdot MAT_{ps}^{2} \cdot MAT_{ss} - 0.00093 \cdot MAT_{ps}^{2} \cdot MAT_{ss}^{2} + 15.792 \cdot LAHM_{ps} - 2.995 \cdot LAHM_{ps}^{2} + 2.061 \cdot LAHM_{ss} - 0.294 \cdot LAHM_{ss}^{2} + 0.021 \cdot SWLNG_{ss} - 0.000058 \cdot SWLNG_{ss}^{2}$$

where LAHMps is a log transformed heat-moisture index at the planting site (set at 2.59 for the current work), LAHMss is a log transformed heat-moisture index at the seed source origin (set at 2.71 for the current work), and SWLNGss is a transformation of seed source latitude and longitude (set at 285.5 for the current work). See Wang et al. (2010) for further details.

MAT_{ss} → / MAT_{ps} ↓	-2	0	2	4	6	8
-2	-0.9	0.2	1.1	1.6	1.7	1.5
0	4.8	5.4	5.7	5.7	5.4	4.7
2	7.7	8.3	8.5	8.4	7.9	7.1
4	7.7	8.8	9.4	9.5	9.3	8.6
6	4.8	6.9	8.3	9.2	9.5	9.3
8	-0.9	2.7	5.5	7.5	8.7	9.1

Figure S1. Response functions for 39 black spruce populations that exhibited a bell-shaped growth response across a mean annual temperature (MAT) gradient. The dashed vertical line indicates MAT at population origin.

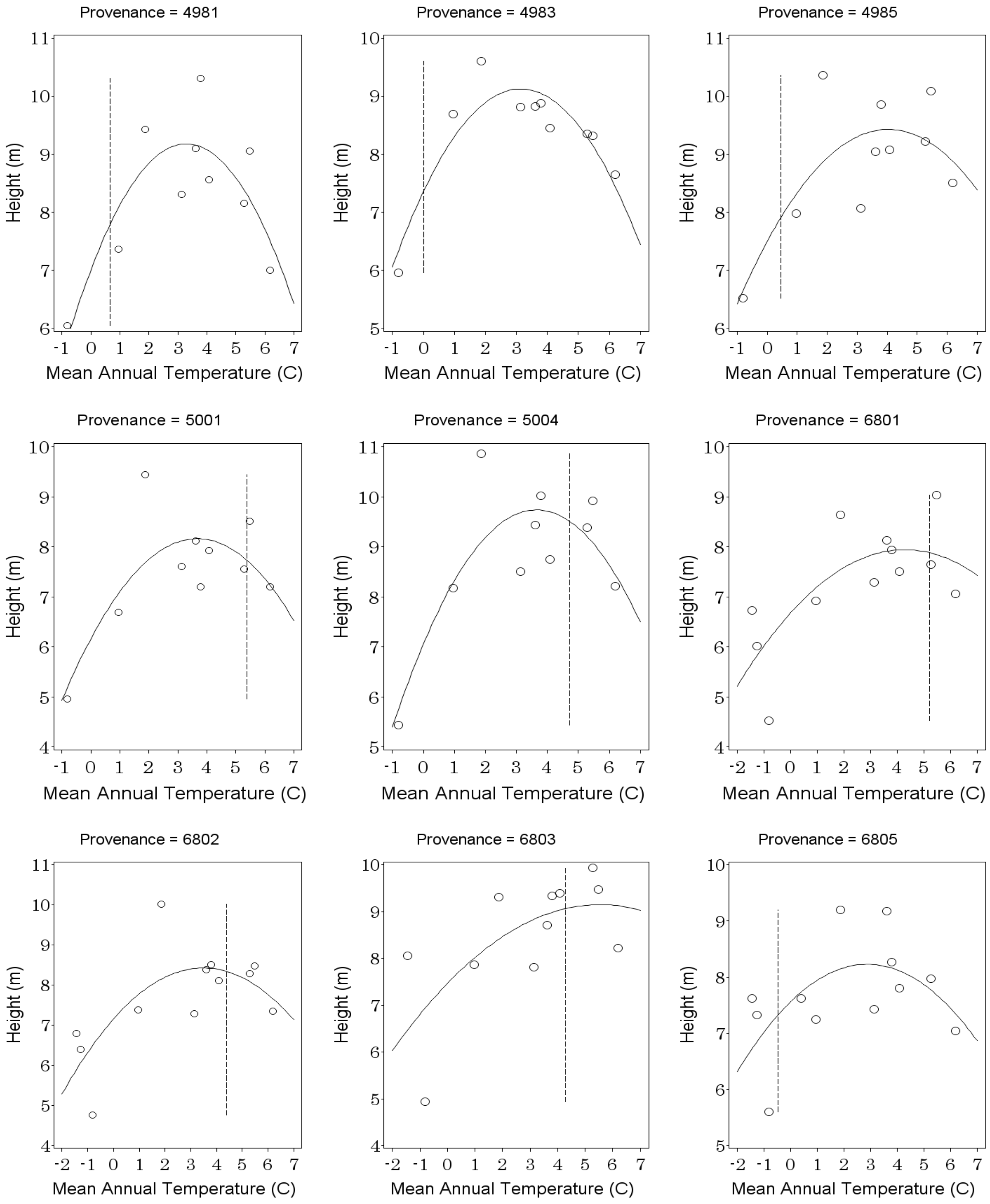

Figure S1. Continued

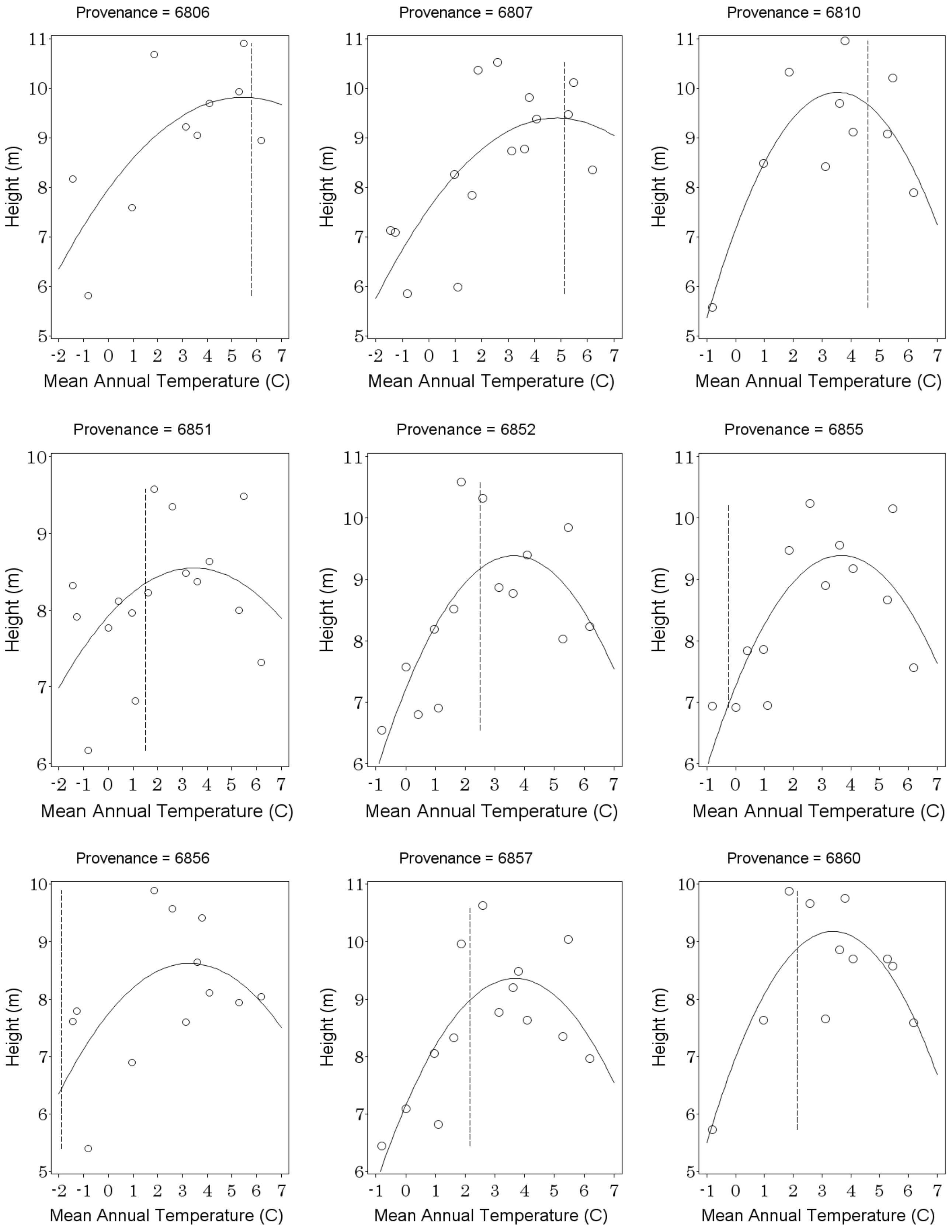

Figure S1. Continued.

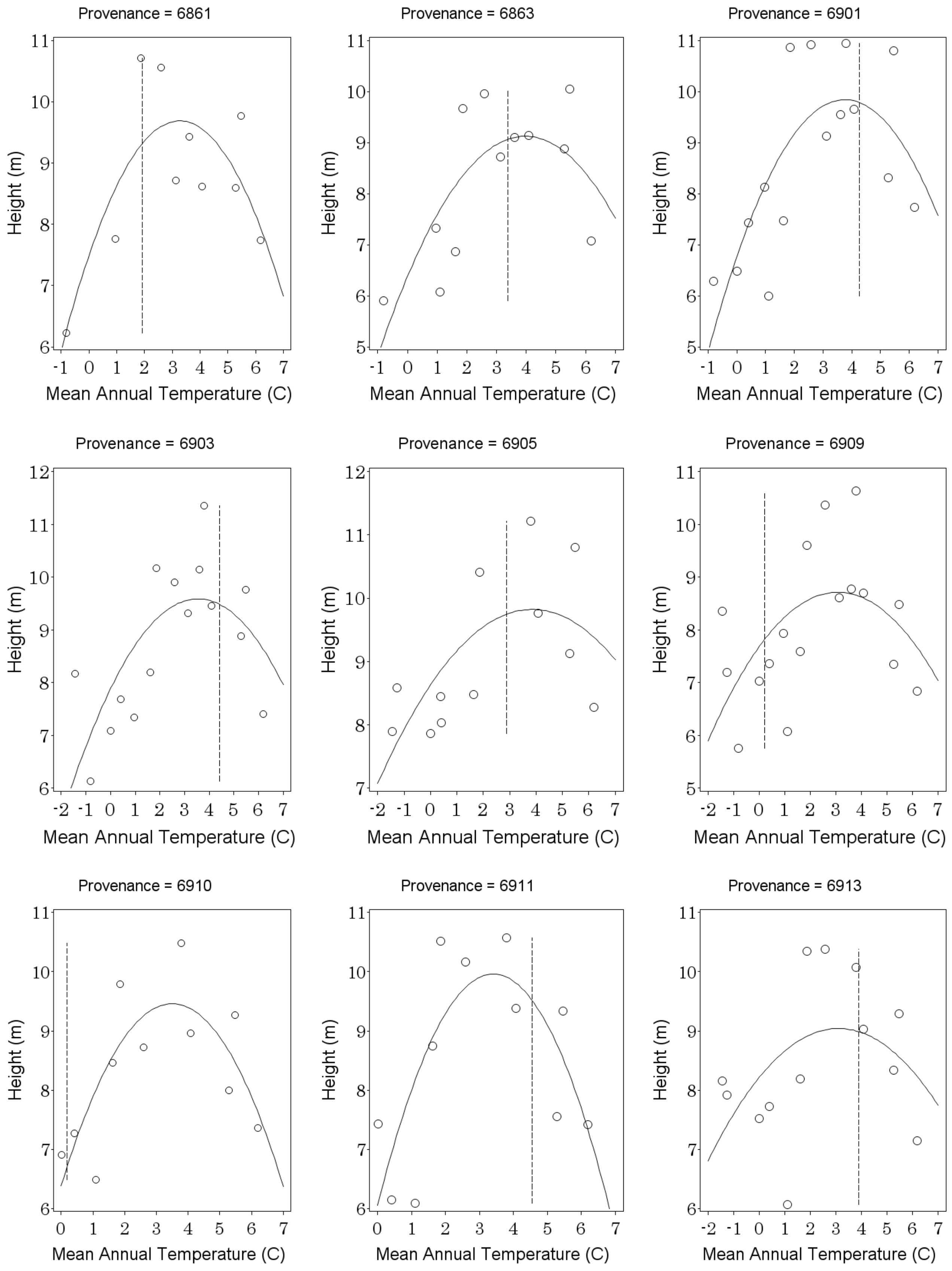

Figure S1. Continued

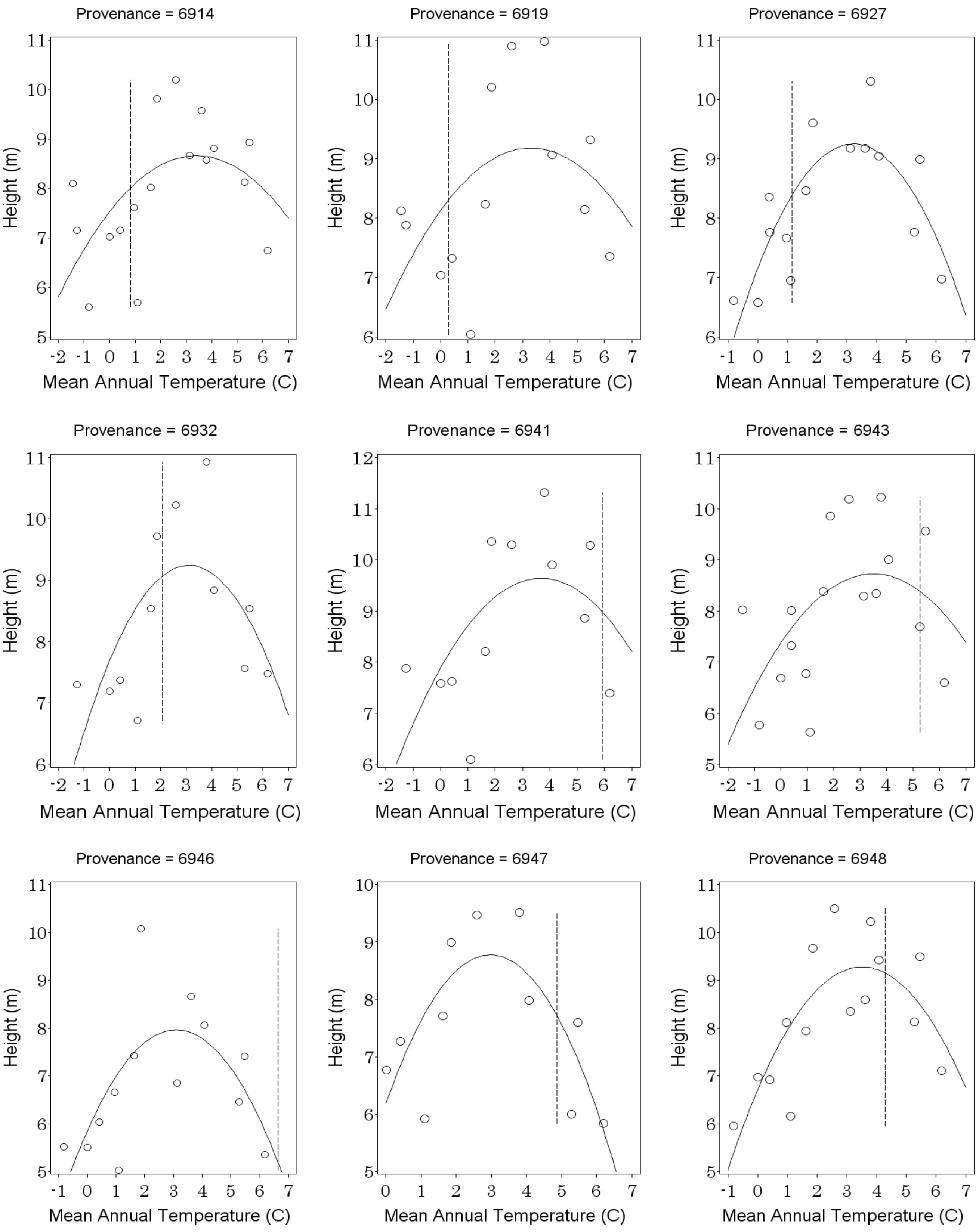

Fig. S1. Continued

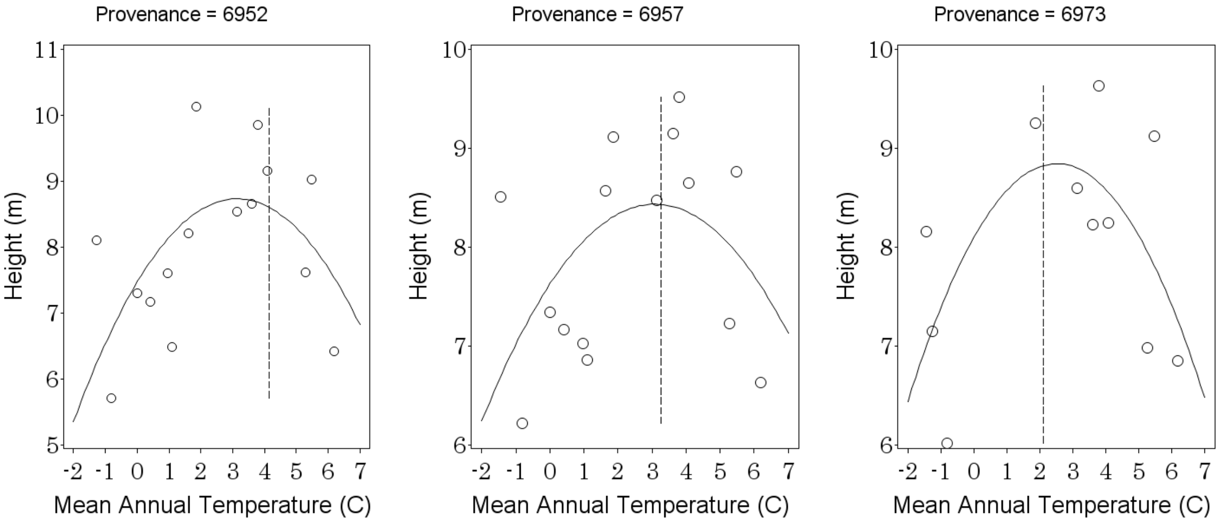

Figure S2. Response functions for 55 jack pine populations that exhibited a bell-shaped growth response across a mean annual temperature (MAT) gradient. The dashed vertical line indicates MAT at population origin.

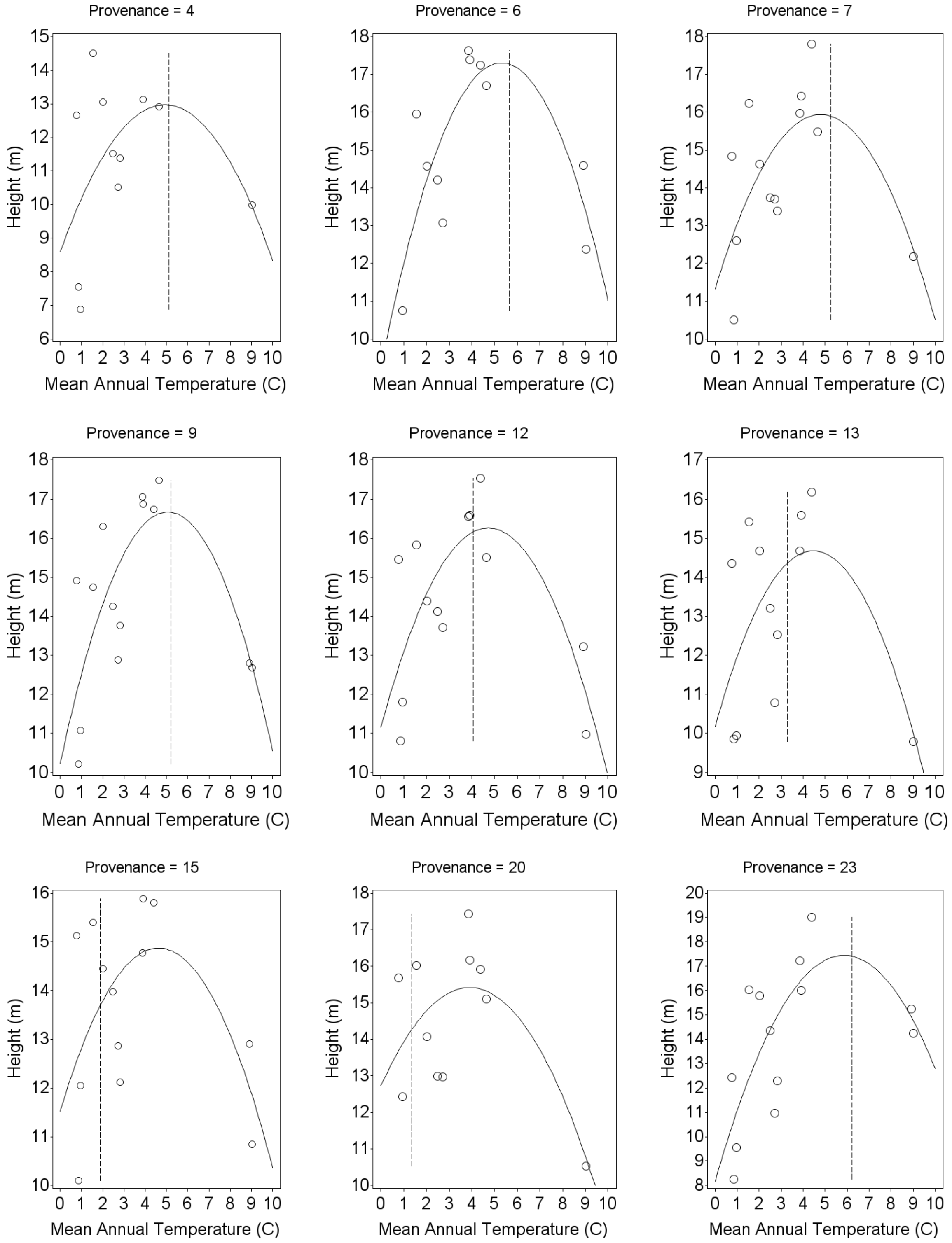

Figure S2. Continued

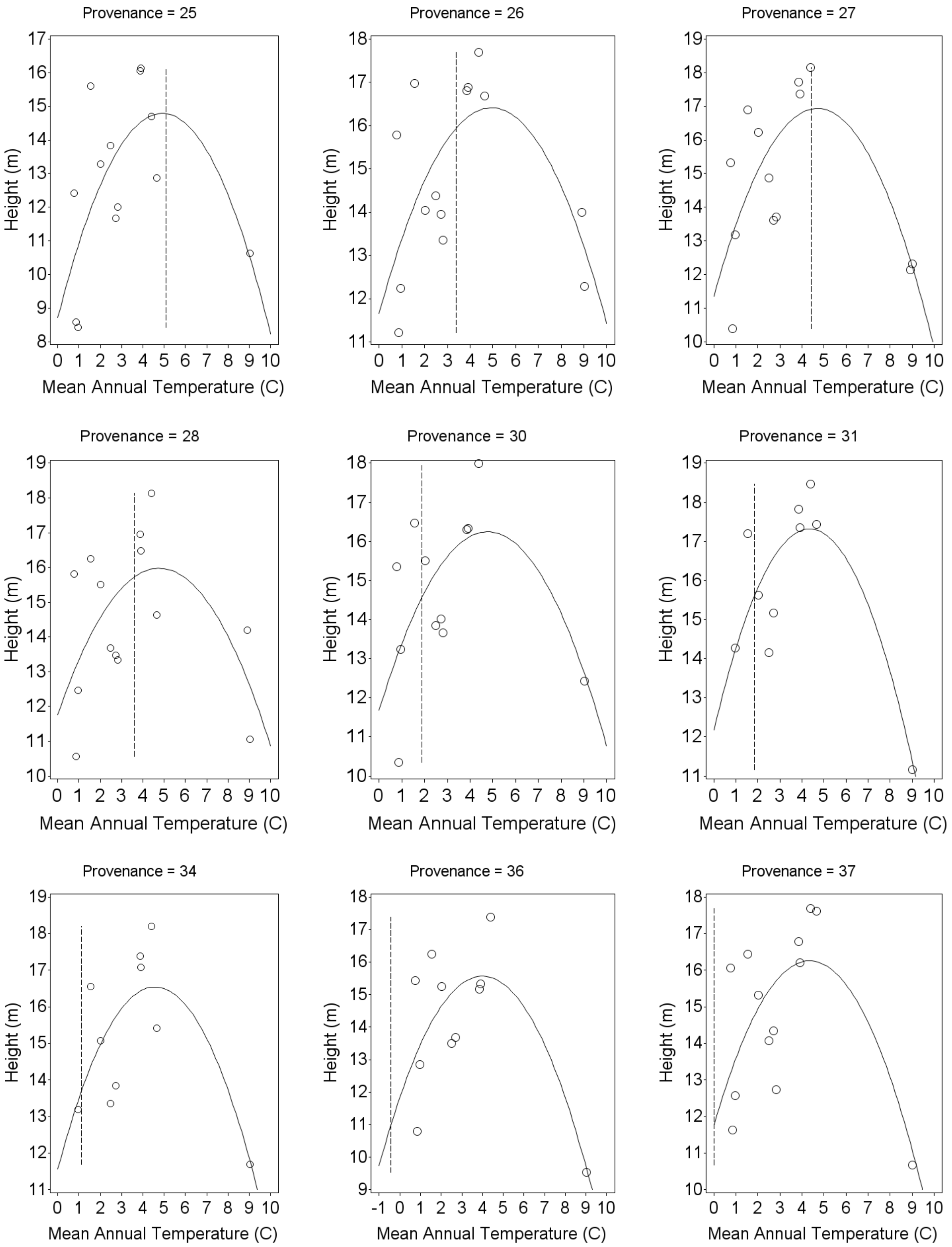

Figure S2. Continued.

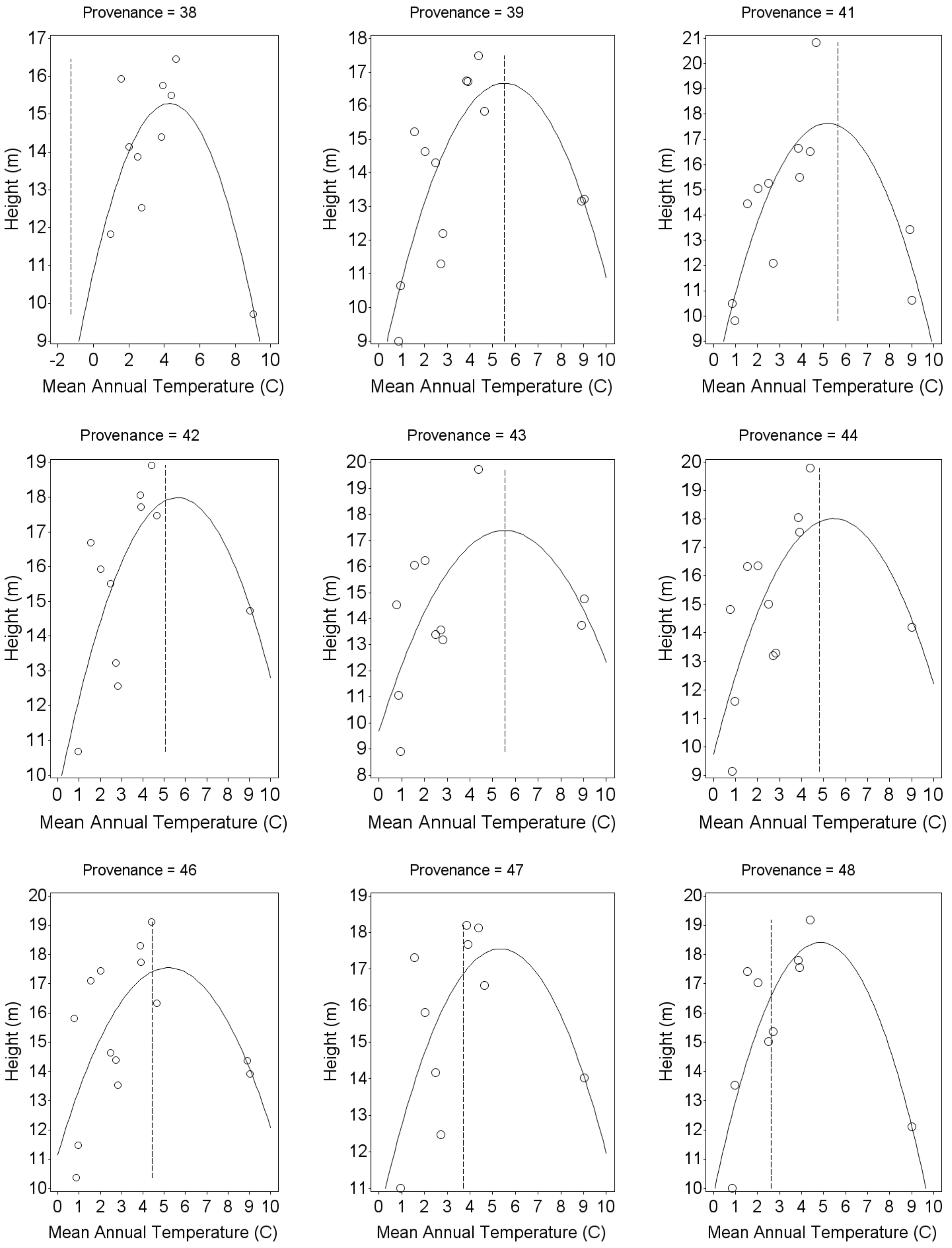

Figure S2. Continued.

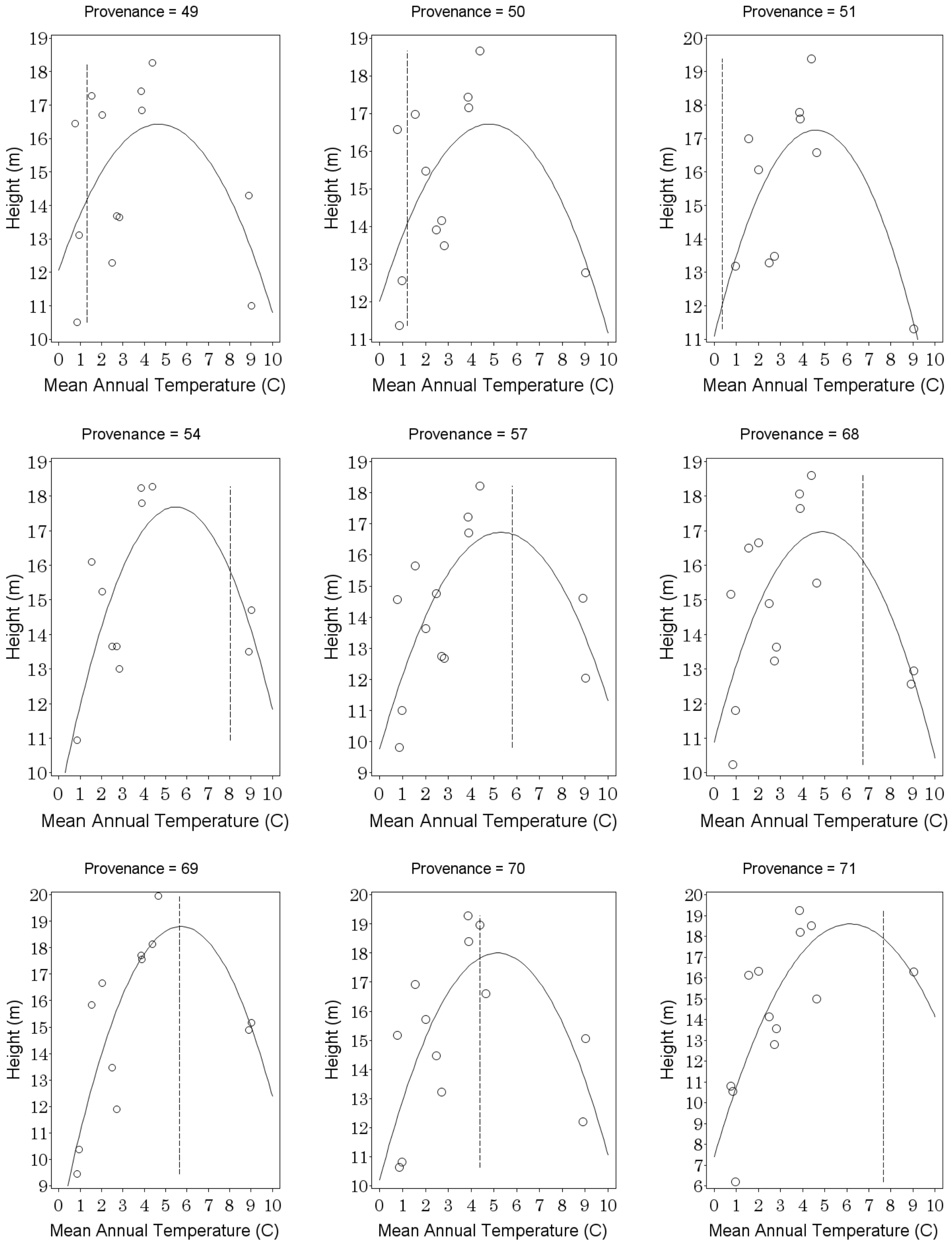

Figure S2. Continued.

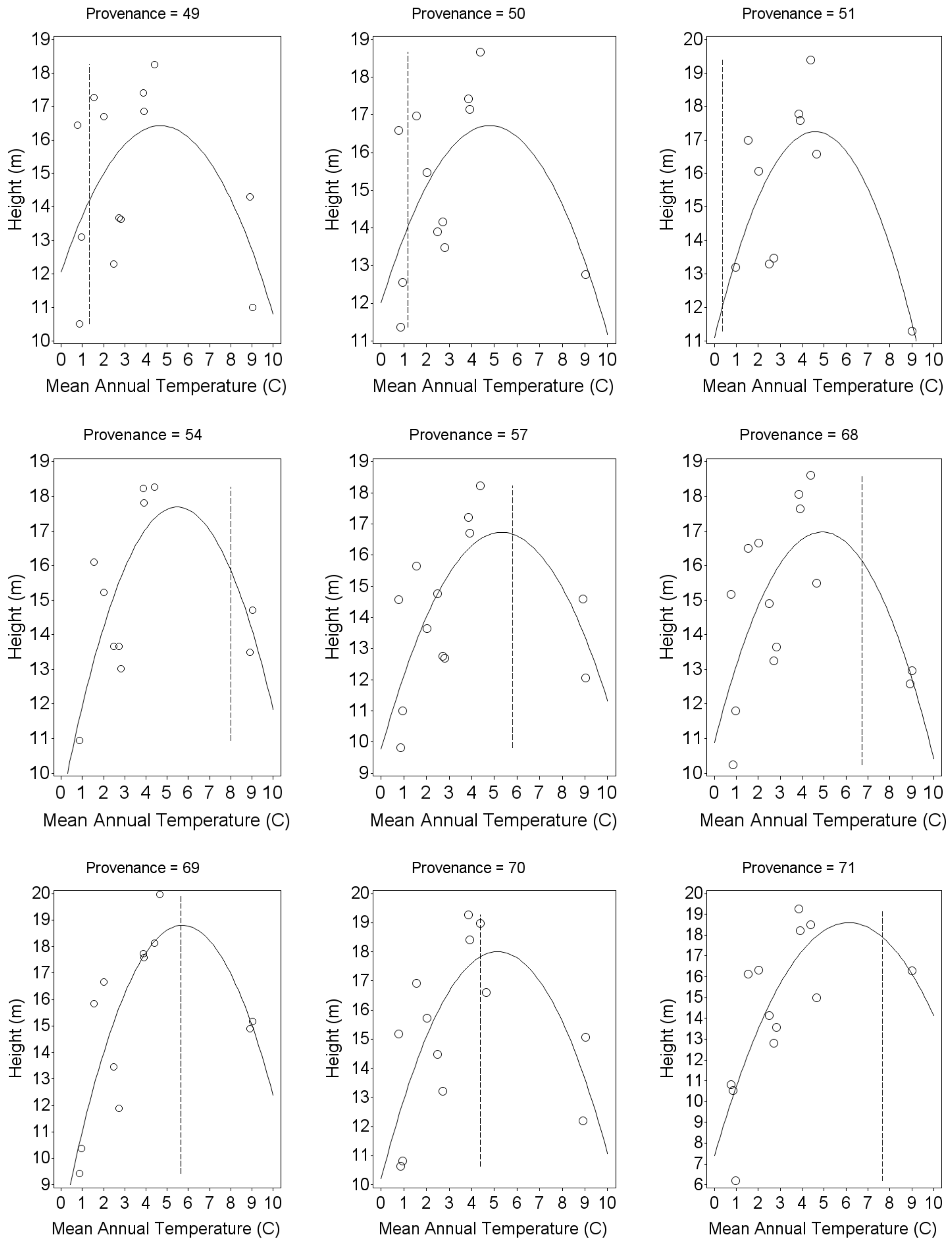

Figure S2. Continued.

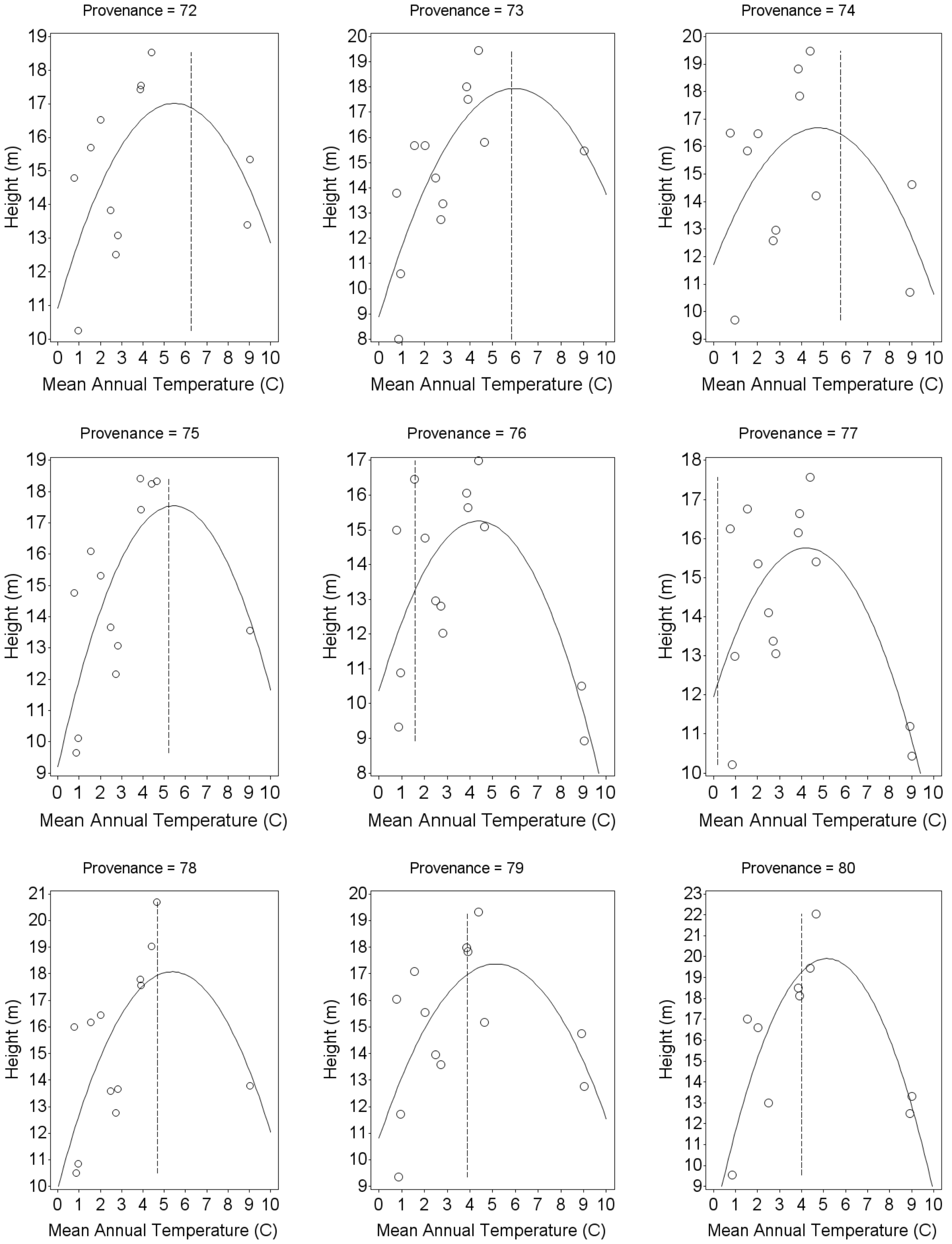

Figure S2. Continued.

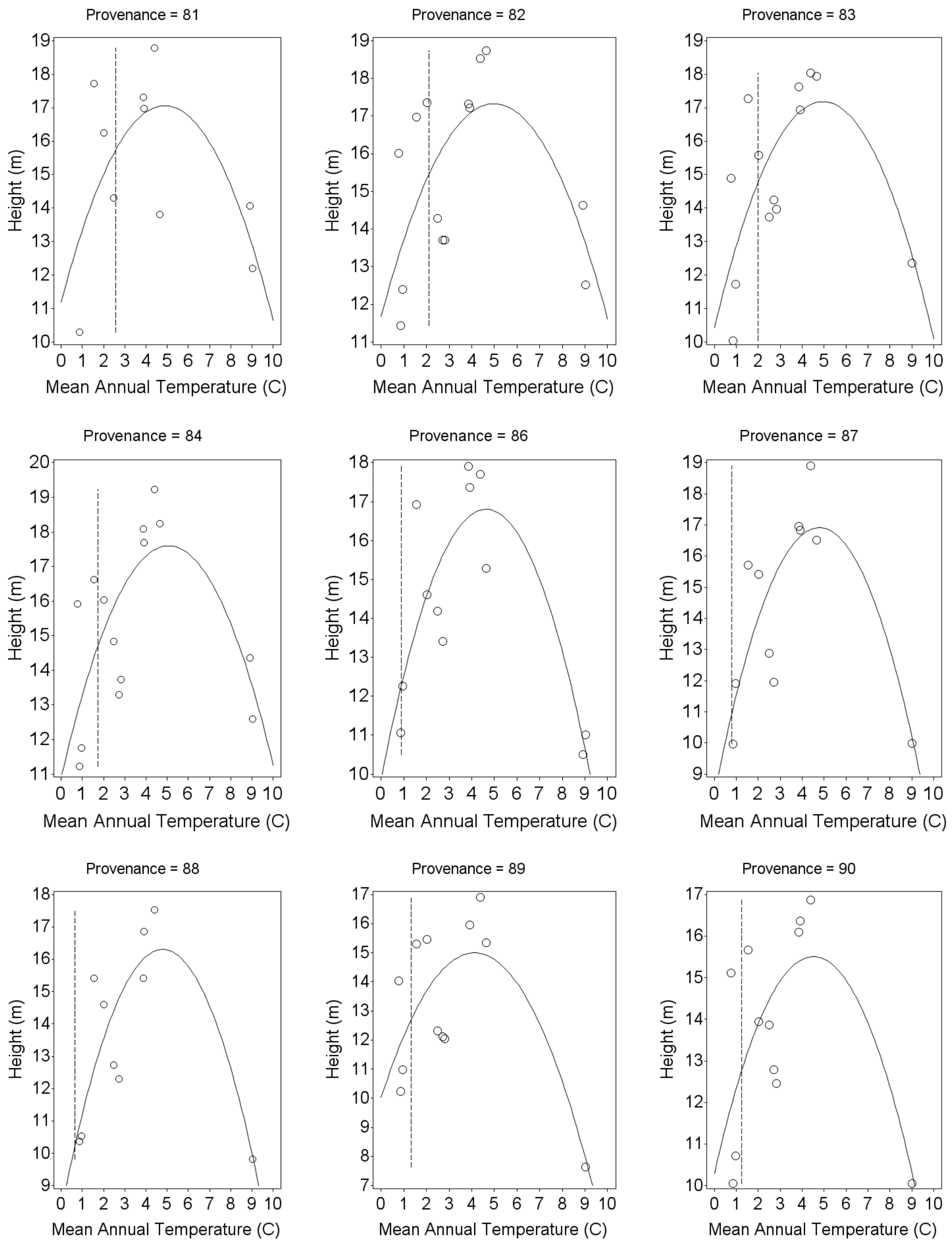

Figure S2. Continued.

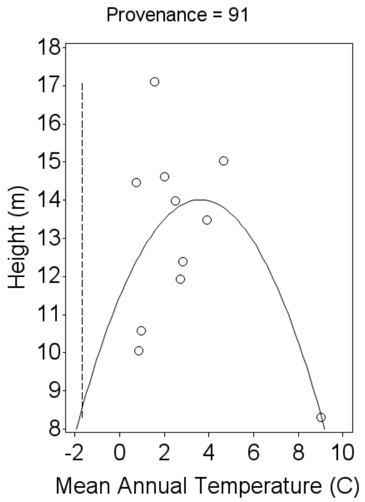

Supplement: Supplementary Material [file srep43881-s1.pdf]
